# Calcitriol Prevents Neuroinflammation and Reduces Blood-Brain Barrier Disruption and Local Macrophage/Microglia Activation

**DOI:** 10.3389/fphar.2020.00161

**Published:** 2020-03-12

**Authors:** Larissa Ragozo Cardoso de Oliveira, Luiza Ayumi Nishiyama Mimura, Thais Fernanda de Campos Fraga-Silva, Larissa Lumi Watanabe Ishikawa, Ana Angélica Henrique Fernandes, Sofia Fernanda Gonçalves Zorzella-Pezavento, Alexandrina Sartori

**Affiliations:** ^1^ Department of Tropical Diseases, Botucatu Medical School, São Paulo State University (UNESP), Botucatu, Brazil; ^2^ Department of Microbiology and Immunology, Institute of Biosciences, São Paulo State University (UNESP), Botucatu, Brazil; ^3^ Department of Chemistry and Biochemistry, Institute of Biosciences, São Paulo State University (UNESP), Botucatu, Brazil

**Keywords:** experimental autoimmune encephalomyelitis, blood-brain barrier, 1,25-dihydroxyvitamin D3, inflammasome, oxidative stress

## Abstract

Multiple sclerosis (MS) is a progressive disease of the central nervous system (CNS) that involves damage to the myelin sheath surrounding axons. MS therapy is based on immunomodulatory drugs that reduce disease recurrence and severity. Vitamin D is a hormone whose immunomodulatory ability has been widely demonstrated, including in experimental autoimmune encephalomyelitis (EAE), which is an animal model of CNS inflammation. In this study, we evaluated the potential of very early intervention with the active form of vitamin D (1,25-dihydroxyvitamin D3) to control neuroinflammation during EAE development. EAE was induced in C57BL/6J mice and 1,25-dihydroxyvitamin D3 administration began 1 day after disease induction. This procedure decreased prevalence, clinical score, inflammation, and demyelination. It also reduced MHCII expression in macrophages and microglia as well as the level of oxidative stress and messenger RNA (mRNA) expression for *NLRP3*, *caspase-1*, *interleukin (IL)-1β, CX_3_CR1, CCL17, RORc and Tbx21* at the CNS. Otherwise, mRNA expression for *ZO-1* increased at the lumbar spinal cord. These effects were accompanied by the stabilization of blood-spinal cord barrier permeability. The results of this study indicate that early intervention with 1,25-dihydroxyvitamin D3 can control the neuroinflammatory process that is the hallmark of EAE and MS immunopathogenesis and should thus be explored as an adjunct therapy for MS patients.

## Introduction

Multiple sclerosis (MS) has been classically categorized as an autoimmune disease in which self-reactive T CD4+ (Th1/Th17), T CD8+, and B cells attack the central nervous system (CNS) and cause neurological dysfunction (Yadav et al., 2015). Although its classification as an autoimmune pathology has been debated (Dendrou et al., 2015), it is widely accepted based on experimental documentation that an autoimmune response to various components of the myelin sheath is a hallmark of this disease (Schultz et al., 2017). It has been postulated that MS starts when self-reactive T cells are activated by autoantigen recognition in peripheral lymphoid organs. Then, by interacting with adhesion molecules and moving according to chemokine gradients (Scheu et al., 2017), these cells cross the blood-brain barrier (BBB) and reach the CNS parenchyma. Once in the CNS, these cells recognize self-antigens at the surface of antigen-presenting cells (APCs), undergo activation and proliferation, and release a plethora of proinflammatory mediators (Garg and Smith, 2015). Together, cells from innate and specific immunity trigger an inflammatory process that culminates in demyelination and axonal loss (Hemme et al., 2015). Increasing evidence indicates that oxidative stress and inflammasome activation serve a major role in demyelination and axonal damage (Ohl et al., 2016; Barclay and Shinohara, 2017).

Despite this presumed sequential pattern, it is well established that MS is clinically heterogeneous and shaped by a complex interaction between genetic background and environmental factors (Waubant et al., 2019). According to Lublin and Reingold (1996), the most common clinical courses in MS patients are relapsing-remitting MS (RRMS), secondary progressive MS (SPMS), primary progressive MS (PPMS), and progressive relapsing MS (PRMS). The frequency of patients that develop each MS course was recently revised by Filippi et al. (2018). According to these authors, RRMS accounts for the majority (approximately 85%) of MS patients and is identified by relapses at variable time intervals. Furthermore, approximately 10–15% of MS patients are affected by the PPMS type, which is distinguished by disease progression from the onset. PRMS is rare and characterized by progression from the onset coupled with acute relapses.

Many disease-modifying therapies for MS are currently approved by the Food and Drug Administration. Most of them are immunomodulatory or immunosuppressive drugs that significantly reduce the frequency of relapses in RRMS. Unfortunately, their effectiveness in avoiding the conversion to the progressive disease remains very limited. Moreover, they are ineffective when the disease has already advanced to the progressive phase (Gajofatto and Benedetti, 2015). There is a growing consensus that the earlier the intervention, the more effective it will be in preventing neurodegeneration (Dobson and Giovanonni, 2019).

Another relevant issue in current MS therapy is the risk of severe side effects, including the possibility of acquiring severe infections (Filippi et al., 2018). These limitations have led to the search for alternative medicines. Epidemiological findings indicating that vitamin D levels were below normal levels in many MS patients (Ghareghani et al., 2018) originated the proposal to treat this disease through vitamin D supplementation. Many of these studies have been performed in rodents with experimental autoimmune encephalomyelitis (EAE), which is the most commonly used model for MS preclinical investigations (Constantinescu et al., 2011; Robinson et al., 2014). Various studies have confirmed that vitamin D is able to control EAE development (Heine et al., 2008; Lysandropoulos et al., 2010; Mayne et al., 2011), with its ability to control MS and EAE being primarily attributed to its immunomodulatory effect and direct activities over the CNS. These effects are mediated by the presence of vitamin D receptor (VDR) and 1α-hydroxylase that catalyzes the production of active vitamin D in macrophages, dendritic cells (DCs), T and B activated lymphocytes, BBB endothelial cells, microglia, and neurons. Vitamin D is well-recognized as a neurosteroid that modulates multiple brain functions. A growing body of evidence indicates that it plays a pivotal role in brain development, neurotransmission, and neuroprotection (DeLuca et al., 2013; Cui et al., 2017). In different neurotoxicity models in rats, vitamin D administration promoted neuron survival by regulating calcium levels and decreasing free radical production. Furthermore, *in vitro* studies using rat neuron cultures demonstrated that active vitamin D (1,25-dihydroxyvitamin D3) increases glutathione levels in these cells. The reduced form of glutathione, which is supplied to nerve cells by astrocytes, is a key antioxidant that protects cells against reactive oxygen species (ROS) and apoptosis (Shinpo et al., 2000; Eyles et al., 2003).

Previous findings from our group indicated that 1,25-dihydroxyvitamin D3 (1,25-VitD3) has a protective effect when given alone or in combination with the specific autoantigen (myelin oligodendrocyte glycoprotein-MOG) (Chiuso-Minicucci et al., 2015; Mimura et al., 2016). Nonetheless, relevant information concerning the local immunopathogenic changes controlled by an early vitamin D administration remains lacking. In this context, we investigated if early intervention with 1,25-VitD3 would be able to control neuroinflammation during EAE development.

## Methods

### Experimental Design

C57BL/6J female mice were allocated into three experimental groups: control (normal animals); EAE (animals immunized with MOG to develop encephalomyelitis), and EAE/Vit D (immunized with MOG and supplemented with 1,25-VitD3). Body weight and clinical scores were evaluated daily, while the tests concerning blood-brain and blood-spinal cord barriers were performed on the 10^th^ day after disease induction. The other assays [histopathological analysis, cell immunophenotyping, oxidative stress status, levels of peripheral cytokine production, messenger RNA (mRNA) expression of chemokines, *ZO-1*, and inflammasome components] were performed at the EAE acute phase, which was 18 days after disease induction. All animals were perfused before collecting the material to be analyzed. All real time polymerase chain reaction (PCR) assays and histopathological analyses were conducted with the lumbar portion of the spinal cord. Oxidative stress was evaluated in homogenates from brain plus cervical and thoracic portions of the spinal cord, and flow cytometry was performed with cells eluted from the whole CNS.

### Animals

Female C57BL/6J mice were bought from the animal facility of the Medical School—University of São Paulo (USP), Ribeirão Preto, São Paulo, Brazil. Water and food were sterilized and offered on an *ad libitum* basis. Animals were housed in ventilated cages (Alesco^®^, Monte Mor, SP, Brazil) in pathogen-free conditions and handled according to the ethical procedures established by the National Council for Control of Animal Experimentation (CONCEA, Brazil). The entire procedure was analyzed and approved by the Ethics Committee on Animal Experimentation of the Institute of Biosciences, UNESP, Botucatu (CEUA-Process 926).

### Experimental Autoimmune Encephalomyelitis Induction, 1,25-Dihydroxyvitamin D3 Administration, and Disease Evaluation

Nine-week-old female C57BL/6J mice were subcutaneously immunized at the base of the tail with 100 μg of MOG_35–55_ (Genemed Synthesis Inc., San Antonio, TX, USA) emulsified with complete Freund's adjuvant (CFA) containing 4 mg/ml of *Mycobacterium tuberculosis* (Difco Laboratories Inc., Detroit, MI, USA). Mice also received two intraperitoneal (IP) doses of *Bordetella pertussis* toxin (Sigma-Aldrich, St. Louis, MO, USA) at 200 ng each: one at the immunization day and the other 48 h after disease induction. Body weight and disease scores were recorded daily. Disease clinical scores were defined according to the following manifestations: no symptoms (0); limp tail (1); hind leg weakness (2); partially paralyzed hind legs (3); complete hind leg paralysis (4); and complete paralysis (5). Percentage of body weight loss was determined between day 0 when the animals were weighed for the first time and submitted to EAE induction and at the end-point of the experiment, i.e. day 18, when they were euthanized and the different parameters were evaluated. A vitamin stock was made by dilution in absolute ethanol. Mice were treated with eight doses (0.1 μg/100 μl of saline containing 15% ethanol) of 1α,25-dihydroxyvitamin D3 (1,25-VitD3—Sigma-Aldrich). This dose corresponds to approximately 5 µg/kg and was delivered by the IP route every other day, as previously described (Chiuso-Minicucci et al., 2015). Supplementation was initiated 24 h after immunization. An EAE group injected only with the vehicle (saline containing 15% ethanol) was initially included to evaluate the influence of the diluent administration on disease development.

### Inflammation and Demyelination

Histopathological evaluations were performed 18 days after disease induction. Lumbar spinal cord segments were fixed in 10% neutral buffered formalin dehydrated in graded ethanol and then included in Paraplast Plus (McCormick, St. Louis, MO, USA). Then, 5 μm-thick sections were obtained using a Leica RM2245 microtome and stained with hematoxylin and eosin (H&E) or luxol fast blue (LFB) to analyze the inflammation and demyelination, respectively. The intensity of the inflammatory infiltration was defined according to the criteria used by other authors (Kluver and Barrera, 1953; Adzemovic et al., 2013; Soellner et al., 2013), which include: absence of inflammatory infiltrate (0); focal meningeal infiltrates (1); more pronounced meningeal infiltrates (2); pronounced meningeal, and some parenchymal infiltration (3).

### Blood-Central Nervous System Barrier Permeability Assay

This evaluation was performed 10 days after disease induction and was based on the NaFlu protocol described by Walker-Caulfield et al. (2015). Briefly, mice were injected *via* the IP route with 100 μl of a 10% sodium fluorescein (NaFlu, Sigma-Aldrich) solution. They were then anesthetized 20 min later and blood was collected for further plasma separation. The animals were then perfused with 10 ml of 0.9% saline solution and the brain and entire spinal cord were collected and individually processed. After being weighed, these organs were macerated with 400 μl of saline solution and then centrifuged (8,600 g, 22°C for 10 min). Supernatants and plasma samples were then distributed in a 96-well black plate and read in a Synergy 4 fluorimeter (BioTek Instruments, Winooski, VT, USA). NaFlu uptake by the brain and spinal cord were calculated according to the following equation: (tissue sample RFU/tissue sample weight)/(plasma RFU/amount of cardiac blood). RFU: relative units of fluorescence.

### Central Nervous System Cells Isolation and Immunophenotyping

Cells from the CNS were obtained according to Pino and Cardona (2011). Briefly, the brain and whole spinal cord were collected after animals’ anesthesia and perfusion. Both organs were initially macerated and further incubated in Roswell Park Memorial Institute (RPMI) medium (Sigma-Aldrich) containing 2.5 mg/ml of collagenase D (Roche Applied Science, Indianapolis, IN, USA) and 100 μg/ml of DNase (Sigma-Aldrich). After incubation (45 min/37°C/CO_2_ 5%), the cells were washed with RPMI and resuspended in a 30% Percoll solution (GE, Healthcare, Uppsala, Sweden). This cell suspension was gently dispensed over 70% Percoll and centrifuged at 950 g for 20 min. The ring containing mononuclear cells was collected, washed, resuspended (1x10^6^ cells/ml), and incubated with the following fluorochrome-labeled monoclonal antibodies (eBioscience, San Diego, CA, USA) according to Murphy et al. (2010): 0.2 μg anti-CD11b-PerCP-Cy5.5 (M1/70), 0.5 μg anti-CD45-FITC (30-F11), 0.03 μg anti-MHCII-APC (MS/114.15.2), 0.15 μg anti-CD40-PE (1C10), and 0.1 μg anti-PD-L1-PE-Cy7 (MIH5) during 30 min and 4°C in the dark. After being washed and resuspended with autoMacs Running Buffer-Miltenyi, the labeled cells were fixed in paraformaldehyde 1% and acquired by using the FACSCanto II flow cytometer (Becton Dickinson, San Jose, CA, USA). The data were analyzed using FlowJo software (TreeStar, Ashland, OR, EUA).

### Cell Culture Conditions and Cytokine Quantification

Inguinal lymph node cells were obtained and adjusted to 2.5x10^5^ cells/ml and then cultured in complete RPMI medium (RPMI supplemented with 10% of fetal calf serum and 2 mM of glutamine). Cultures were incubated during 48 h in the presence of MOG (20 μg/ml), as previously described by Zorzella-Pezavento et al. (2017). Cytokine levels were then quantified by enzyme-linked immunosorbent assay (ELISA) in culture supernatants using IFN-γ BD OptEIA Sets (BD Biosciences, San Diego, CA, USA) and interleukin (IL)-6, TNF-α, and IL-17 DuoSets (R&D Systems, Minneapolis, MN, USA). The assays were performed according to the manufacturer’s instructions.

### Oxidative Stress Analysis

Oxidative and anti-oxidative parameters were measured at the CNS using the biochemical methodology previously described by Romualdo et al. (2017). Approximately 500 mg samples of CNS (brain plus cervical and thoracic spinal cord) were mechanically homogenized in sodium phosphate buffer (0.1 M/pH 7.0). This material was centrifuged (1,200 g/15 min) and supernatants used for biochemical assays. Lipid hydroperoxide levels were assessed by an Fe_2_^+^ oxidation reaction in the presence of xylenol orange, sulfuric oxide, and butylated hydroxytoluene diluted in methanol. To evaluate the degree of protein carbonylation, the samples were incubated with 2,4-dinitrophenylhydrazine and centrifuged in the presence of trichloroacetic acid. The protein pellet was washed with an ethanol-ethyl acetate mixture and then resuspended in guanidine hydrochloride. Catalase activity was evaluated by sample incubation with hydrogen peroxide diluted in phosphate buffer, and glutathione peroxidase activity was determined by a NADPH_2_ oxidation reaction in the presence of hydrogen peroxide and glutathione reductase. Superoxide dismutase activity was determined in a pH10 medium by measuring the inhibitory enzymatic activity on nitro-blue tetrazolium reduction triggered by hydroxylamine-derived free radicals. Quantification of the aforementioned oxidative stress parameters was based on absorbances detected by a microplate reader (mQuant-Gen5 2.0 software, BioTek).

### Messenger RNA Expression of Inflammasome Components, T Cell Transcription Factors, *CX3CR1*, *CCL17*, and *ZO-1*

Total RNA was extracted from the lumbar spinal cord (TRIzol reagent, Life Technologies, Carlsbad, CA, USA) and complementary DNA (cDNA) was synthesized using the Kit High Capacity (Applied Biosystems, Foster City, CA, USA). *NLRP3*, *ASC*, *Caspase-1*, *IL-1β*, *CX_3_CR1*, *CCL17*, *RORc*, *GATA3*, *Foxp3*, *Tbx21*, and *GAPDH* gene amplifications were performed using the TaqMan Master Mix with primers and probes inventoried and tested by Life Technologies. *ZO-1* and *GAPDH* gene amplifications were performed by using the Power SYBR Green PCR Master Mix (Applied Biosystems). Primer sequences included: *ZO-1* forward—GAGAGACAAGATGTCCGCCA, reverse—CCATTGCTGTGCTCTTAGCG (NM_001163574.1), and *GAPDH* forward—GCATCTTCTTGTGCAGTGCC, reverse—TACGGCCAAATCCGTTCAC (NM_008084.3). The amplification of specific transcripts was confirmed by the melting curve profiles generated at the end of each run. Assays were performed in duplicate in “Sequence Detection Systems” 1.2.3—7300 Real-Time PCR Systems (Applied Biosystems). The results were expressed based on fold difference using the delta delta cycle threshold (2^−ΔΔCt^) method for quantification with the negative control group as a calibrator, according to Livak and Schmittgen (2001).

### Statistical Analysis

Results were expressed as mean ± standard deviation or as median (25–75%). Comparisons between two samples were made by a t-test and comparisons between more than three samples were made by one-way analysis of variance (ANOVA) followed by Tukey’s test to compare parametric findings. A Kruskal-Wallis test followed by Dunn’s test was employed to analyze and compare non-parametric findings. Disease prevalence was evaluated by the chi-squared test. Statistical analyses were performed using SigmaPlot 12.0 for Windows (Systat Software Inc., San Jose, CA, USA), and p < 0.05 was considered significant. The difference between clinical scores was analyzed by the area under the curve method.

## Results

### Early Intervention With 1,25-VitD3 Controlled Clinical Experimental Autoimmune Encephalomyelitis Development

**Figure 1:**
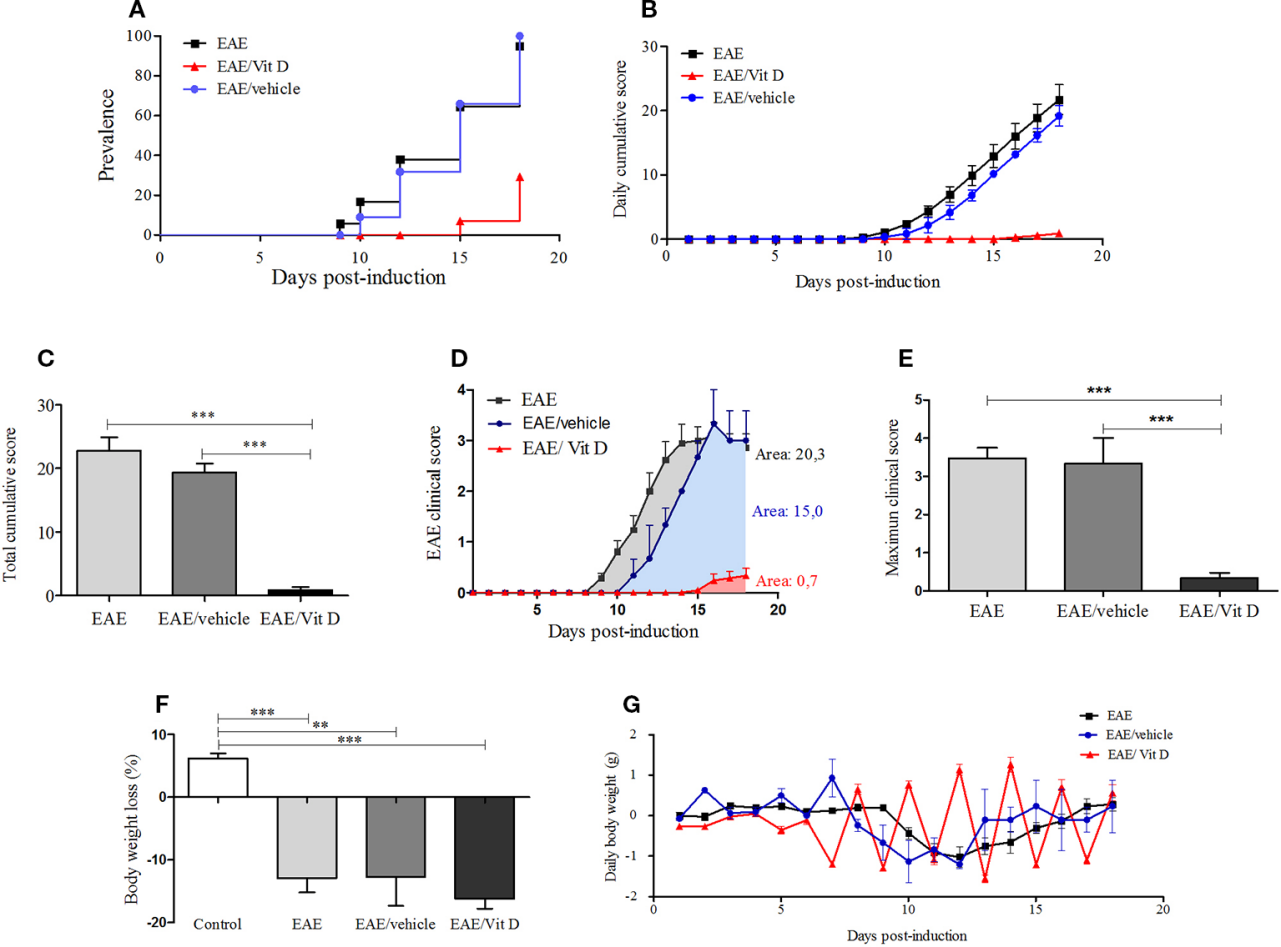
1,25-dihydroxyvitamin D3 decreases experimental autoimmune encephalomyelitis (EAE) prevalence and severity. Encephalomyelitis was induced by immunization with MOG and CFA and two doses of pertussis toxin. Disease prevalence **(A)**, daily cumulative score **(B)**, total cumulative score **(C)**, clinical score **(D)**, maximum clinical score **(E)**, **(F)** and body weight variation **(G)** were analyzed daily from day 1 to day 18. Results were expressed as media ± SEM (control, EAE, and EAE/Vit D group: 21 animals; EAE/vehicle group: three animals). Results from four independent experiments were pooled. ANOVA, Tukey's test **p < 0.01, and ***p < 0.001.

C57BL/6J mice submitted to EAE induction were injected with eight 1,25-VitD3 doses every other day *via* the IP route. This intervention, which began 24 h after disease induction, significantly reduced EAE prevalence (**Figure 1A**). As expected, most animals (95.2%) from the EAE group developed signs of paralysis, whereas only 23.8% of the EAE/Vit D became sick. 1,25-VitD3 also delayed disease onset by 6 days. Daily and total cumulative score, clinical score, and maximum clinical score, which are widely used parameters for evaluating disease severity, were also clearly reduced by this vitamin, as presented in **Figures 1B, C** (mean ± SEM): EAE: 22.8 ± 2.1, EAE/vehicle: 19.3 ± 1.4 and EAE/Vit D: 0.9 ± 0.4; *f*: 56.8, **Figures 1D, E** (mean ± SEM): EAE: 3.5 ± 0.3, EAE/vehicle: 3.3 ± 0.7, and EAE/Vit D: 0.3 ± 0.14; *f*: 53.9, respectively. As expected, EAE animals presented a significant percentage of body weight loss (**Figure 1F**). As illustrated in **Figure 1G**, this body weight loss coincided with disease appearance. Although EAE/Vit D mice were protected from disease, they also presented significant body weight loss (mean ± SEM): control: +6.2 ± 0.8, EAE: −12.9 ± 2.2, EAE/vehicle: −16.2 ± 1.6, and EAE/Vit D: −12.7 ± 4.6; *f*: 29.5 (**Figure 1F**). Distinct from the EAE group, their body weight loss was independent of the disease; that is, the loss began earlier, remained after the acute disease phase, and peaked soon after each vitamin dose (**Figure 1G**). Moreover, disease development was unaffected by the administration of the diluting agent (saline containing 15% ethanol).

### 1,25-VitD3 Attenuated Inflammation and Demyelination at the Spinal Cord

**Figure 2:**
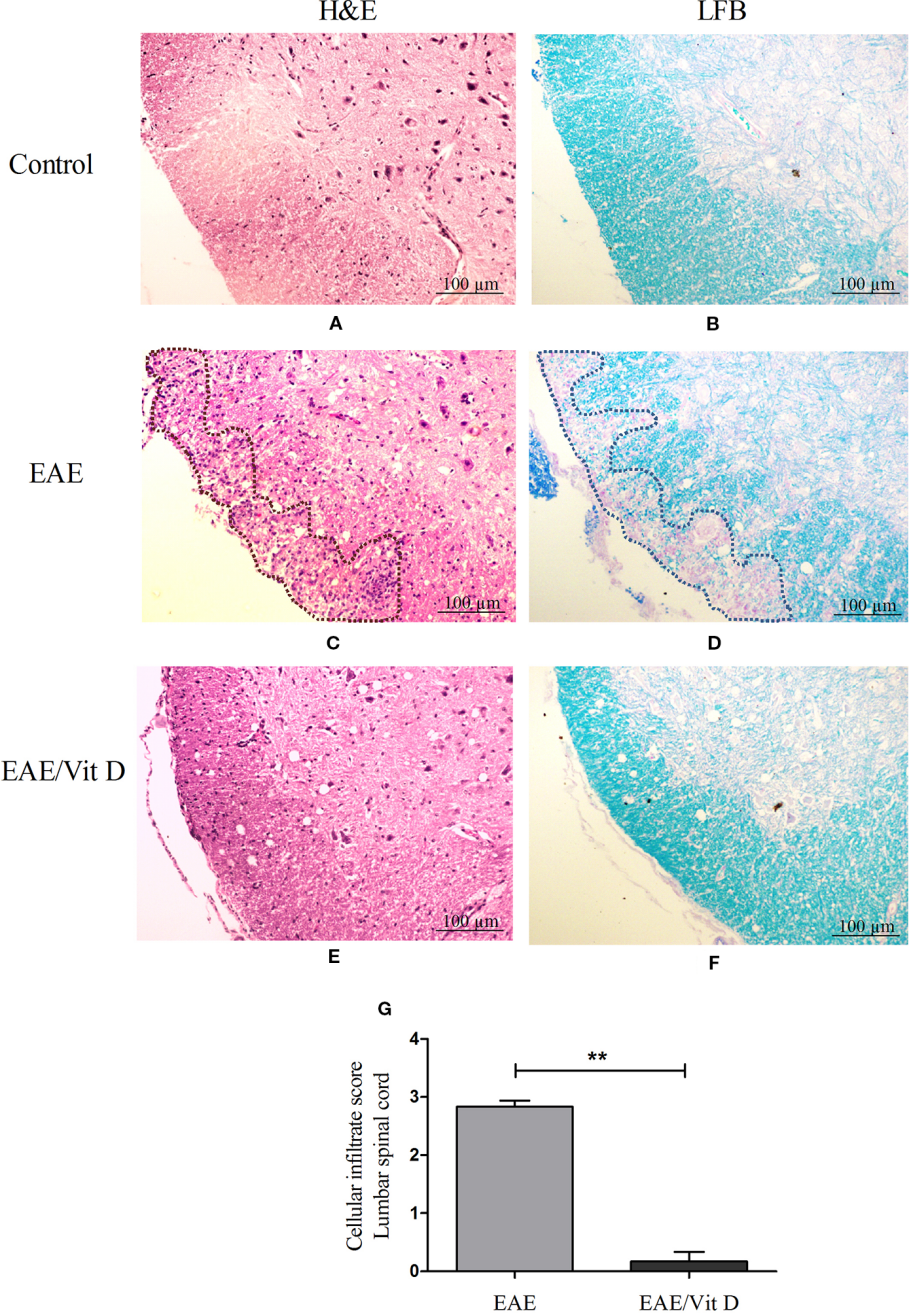
1,25-dihydroxyvitamin D3 reduces inflammation and demyelination at the lumbar spinal cord. Animals from control (normal), EAE and EAE/Vit D groups were anesthetized and underwent perfusion on the 18^th^ day after disease induction (acute phase). The lumbar spinal cord was collected and processed for histopathological analysis. Then, 5 µm sections were stained with H&E or LFB for analysis of the degree of inflammation and demyelination, respectively (scale bar: 100 μm). Representative micrographs of the degree of inflammation in control **(A)**, EAE **(C)**, and EAE/Vit D **(E)** experimental groups. Representative micrographs of the degree of demyelination in control **(B)**, EAE **(D)**, and EAE/Vit D **(F)** groups. The semi-quantitative analysis of the inflammatory process was performed according to the following criteria: absence of inflammatory infiltrate (0); focal meningeal infiltrates (1); more pronounced meningeal infiltrates (2); pronounced meningeal and some parenchymal infiltration (3). Results were expressed as media ± SEM (six animals/group) **(G)**. Results from two independent experiments were combined. ANOVA, Tukey's test **p < 0.01.

H&E-stained lumbar spinal cord sections from EAE mice revealed a conspicuous inflammatory infiltrate located in the meninges and parenchyma. The distribution of the infiltrate is delimited by a dotted line in **Figure 2C**. This is in clear contrast with the findings in EAE/Vit D mice, which showed no inflammation or only scanty inflammatory cells (**Figure 2E**). This differential degree of inflammation was estimated by a semi-quantitative analysis that reinforced the significantly reduced inflammation in EAE/Vit D mice, as depicted in **Figure 2G** (mean ± SEM): EAE: 2.8 ± 0.1 and EAE/Vit D: 0.2 ± 0.2; *f*: 195. LFB-stained sections exhibited demyelinating lesions coinciding with the location of the inflamed region in EAE mice (**Figure 2D**) and their absence in EAE/VitD animals (**Figure 2F**). The expected absence of inflammation and demyelination in spinal cord sections from normal control mice is exhibited in **Figures 2A**, **B**, respectively.

### 1,25-VitD3 Restrained Cell Infiltration Into the Central Nervous System

**Figure 3:**
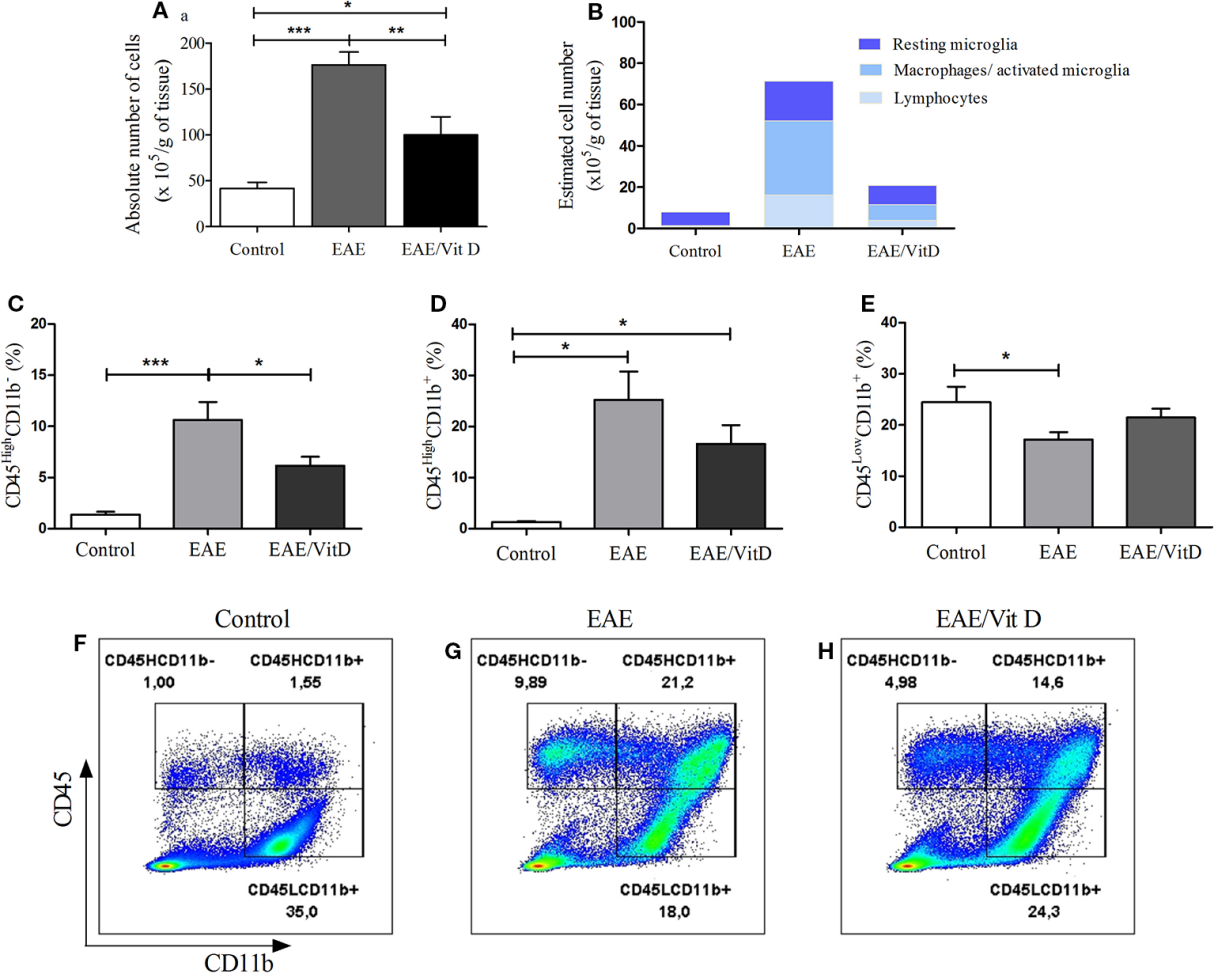
1,25-dihydroxyvitamin D3 reduces the presence of inflammatory cells at the CNS. Brain and spinal cord samples were collected from control, EAE, and EAE/Vit D mice at the acute disease phase (18^th^ day). Eluted cells were counted and analyzed by flow cytometry. Absolute cell number **(A)**; estimated cell number **(B)**; percentage of CD45HighCD11b- **(C)**; percentage of CD45HighCD11b+ **(D)**; percentage of CD45LowCD11b+ **(E)**. Representative dot-plots for **C**–**E** results are shown in **F**–**H**. Results were expressed as media ± SEM (9–15 animals/group). Results from three independent experiments were combined. ANOVA, Tukey's test *p < 0.05, **p < 0.01, and ***p < 0.001.

The total number of cells eluted from the CNS was calculated per gram of tissue and indicated a significant reduction in EAE/Vit D mice (mean ± SEM): control: 41.4 ± 7.1, EAE: 176.2 ± 14.2, and EAE/Vit D: 100.0 ± 19.6; *f*: 21.6 (**Figure 3A**). To elucidate whether this effect targeted particular cell types, the cells were analyzed by flow cytometry. This reduction involved lymphocytes (CD45^High^CD11b^−^) (mean ± SEM): control: 1.3 ± 0.3, EAE: 10.6 ± 1.8, and EAE/Vit D: 6.1 ± 0.9; *f*: 9.9, infiltrating macrophages and activated microglia (CD45^High^CD11b^+^) (mean ± SEM): 1.34 ± 0.2, EAE: 25.2 ± 5.5, and EAE/Vit D: 16.1 ± 3.6; *f*: 9.9, as shown in **Figures 3C**, **D**, respectively. The frequency of resting microglia (CD45^Low^CD11b^+^) (mean ± SEM): control: 24.5 ± 3.0, EAE: 17.2 ± 1.4, and EAE/Vit D: 21.5 ± 1.7; *f*: 3.5, which was predictably lower in EAE animals, was similar to the frequency found in normal mice (**Figure 3E**). To estimate the total cell number of these three cell populations, we used the rule of three mathematical method considering the total number of cells shown in **Figure 3A** being 100%. This calculation, which is illustrated in **Figure 3B**, allowed us to estimate the total number of each cell population (lymphocytes, macrophages/activated microglia, and resting microglia). A striking reduction can be observed in the three populations in the CNS of the EAE/Vit D group. The gate strategy adopted for each experimental group is illustrated in **Figures 3F–H**.

**Figure 4:**
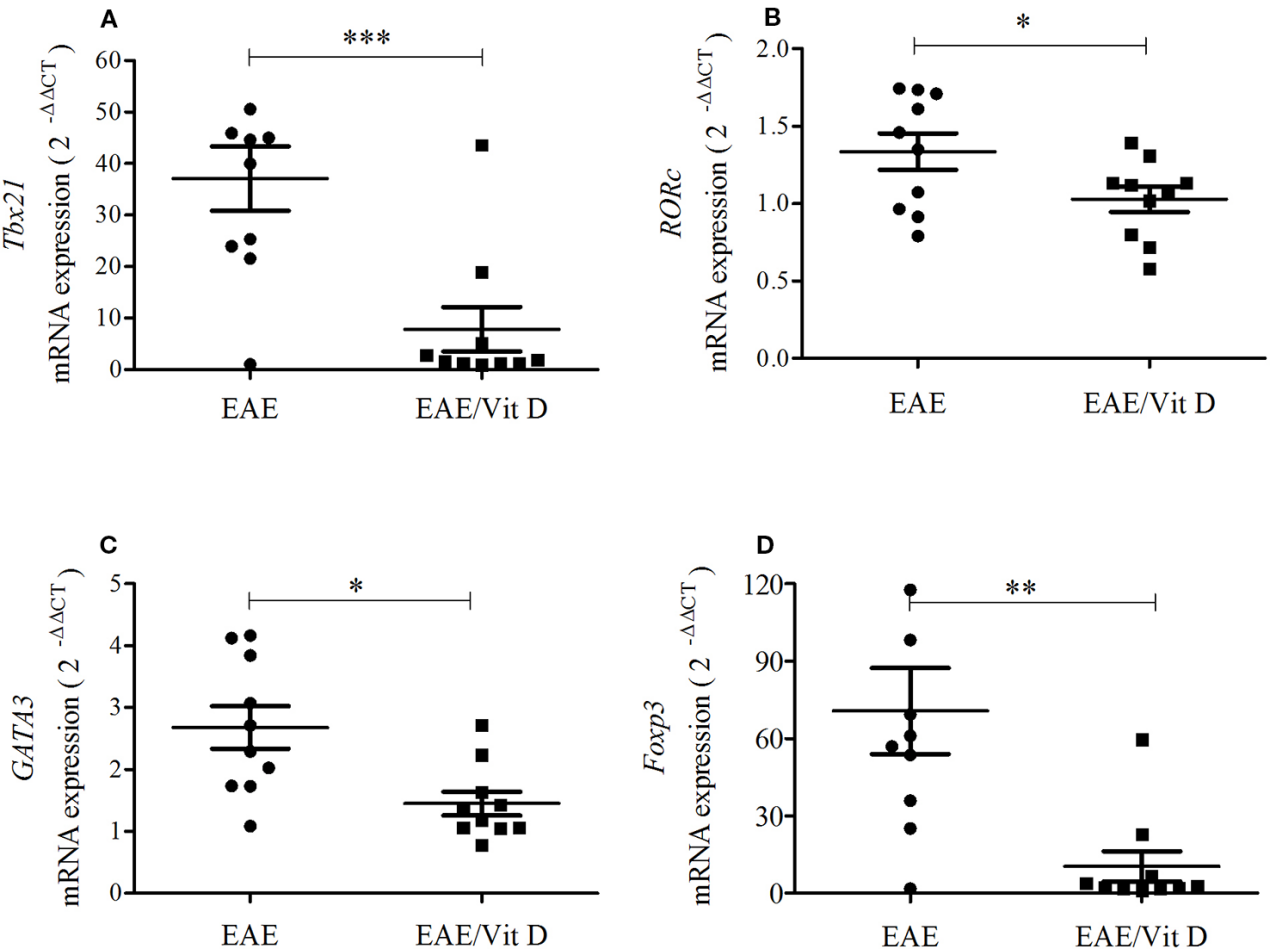
1,25-dihydroxyvitamin D3 reduces the presence of Th subsets at the lumbar spinal cord. The lumbar spinal cord was collected from control, EAE, and EAE/Vit D mice at the acute disease phase (18^th^ day). Total RNA was extracted and the expression levels of mRNA for *Tbx21* **(A)**, *RORc* **(B)**, *GATA3* **(C)** and *Foxp3* **(D)** were established by real-time PCR. The results are expressed as median, 25–75% (box), and minimum-maximum (error bars) (10 animals/group). Results from two independent experiments were combined. Kruskal-Wallis, Dunn's test *p < 0.05, **p < 0.01, and ***p < 0.001.

As lymphocytes were decreased in the CNS of animals that received 1,25-VitD3 and since it is well established that there are T cell subsets specialized in mediating or regulating the immune response, we analyzed the local expression of mRNA for key transcription factors. *Tbx21* and *RORc*, which are expressed by Th1 and Th17 subsets, respectively, were significantly downmodulated in mice supplemented with 1,25-VitD3 (**Figures 4A, B**). These animals also expressed lower levels of *GATA3* (**Figure 4C**) and *Foxp3* (**Figure 4D**) mRNA, which are expressed by Th2 and Tregs, respectively.

### Central Nervous System Inflammatory Parameters Were Downmodulated by 1,25-VitD3 Administration

**Figure 5:**
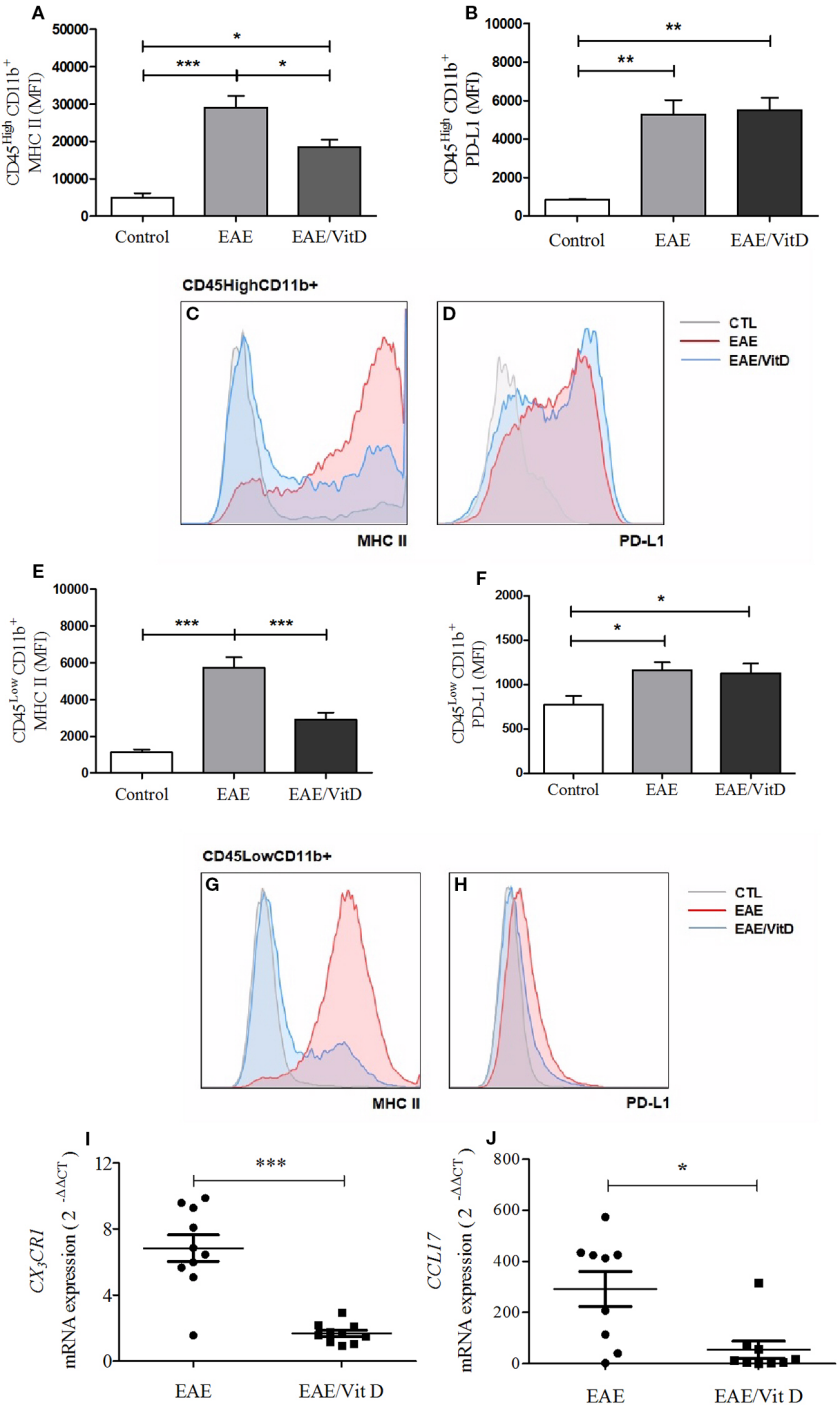
1,25-dihydroxyvitamin D3 downmodulates cellular activity at the CNS. The CNS or the lumbar spinal cord were collected from control, EAE, and EAE/Vit D mice at the acute disease phase (18^th^ day). The entire CNS was processed and analyzed by flow cytometry. MFI associated with MHCII and PD-L1 were evaluated in CD45HighCD11b+ and CD45LowCD11b+. MHCII **(A)** and PD-L1 **(B)** MIF were associated with CD45HighCD11b+ cells and respective representative histograms **(C, D)**. MHCII **(E)** and PD-L1 **(F)** MIF were associated with CD45LowCD11b+ cells and respective representative histograms **(G, H)**. Total mRNA was extracted from the lumbar portion of the spinal cord and the expression levels of *CX3CR1* **(I)** and *CCL17* **(J)** were established by real-time PCR. MFI was expressed as media ± SEM (9–15 animals/group). Results from three independent experiments were combined. ANOVA, Tukey's test *p < 0.05, **p < 0.01, and ***p < 0.001. The results of qPCR are expressed as median, 25–75% (box), and minimum-maximum (error bars) (10 animals/group). Results from two independent experiments were combined. Kruskal-Wallis, Dunn's test *p < 0.05, **p < 0.01, and ***p < 0.001.

The inflammatory process that occurs in the CNS during EAE development involves, among other mechanisms, increased MHCII expression, the contribution of chemokines and their receptors, oxidative stress, and the activation of the inflammasome platform. 1,25-VitD3 downmodulated all of these parameters, which were clearly elevated in EAE mice. 1,25-VitD3 decreased the MHCII mean fluorescence intensity (MFI) in the cell population constituted by macrophages and activated microglia (mean ± SEM): control: 4,977 ± 1,118, EAE: 29,060 ± 3,091, and EAE/Vit D: 18,510 ± 1,959; *f*: 13.3 (**Figures 5A, C**) and in resting microglia (mean ± SEM): control: 1,118 ± 165, EAE: 5,718 ± 572, and EAE/Vit D: 2,900 ± 390; *f*: 21.2 (**Figures 5E, G**). No alterations were detected in PD-L1 expression, as shown in **Figures 5B, D, F, H**.

The local expression of *CX_3_CR1* mRNA (a receptor for fractalkine) and of *CCL17* mRNA (a chemokine involved in leukocyte trafficking) were also significantly downmodulated in mice that received 1,25-VitD3. *CX_3_CR1* and *CCL17* mRNA levels are presented in **Figures 5I**, **J**, respectively.

**Figure 6:**
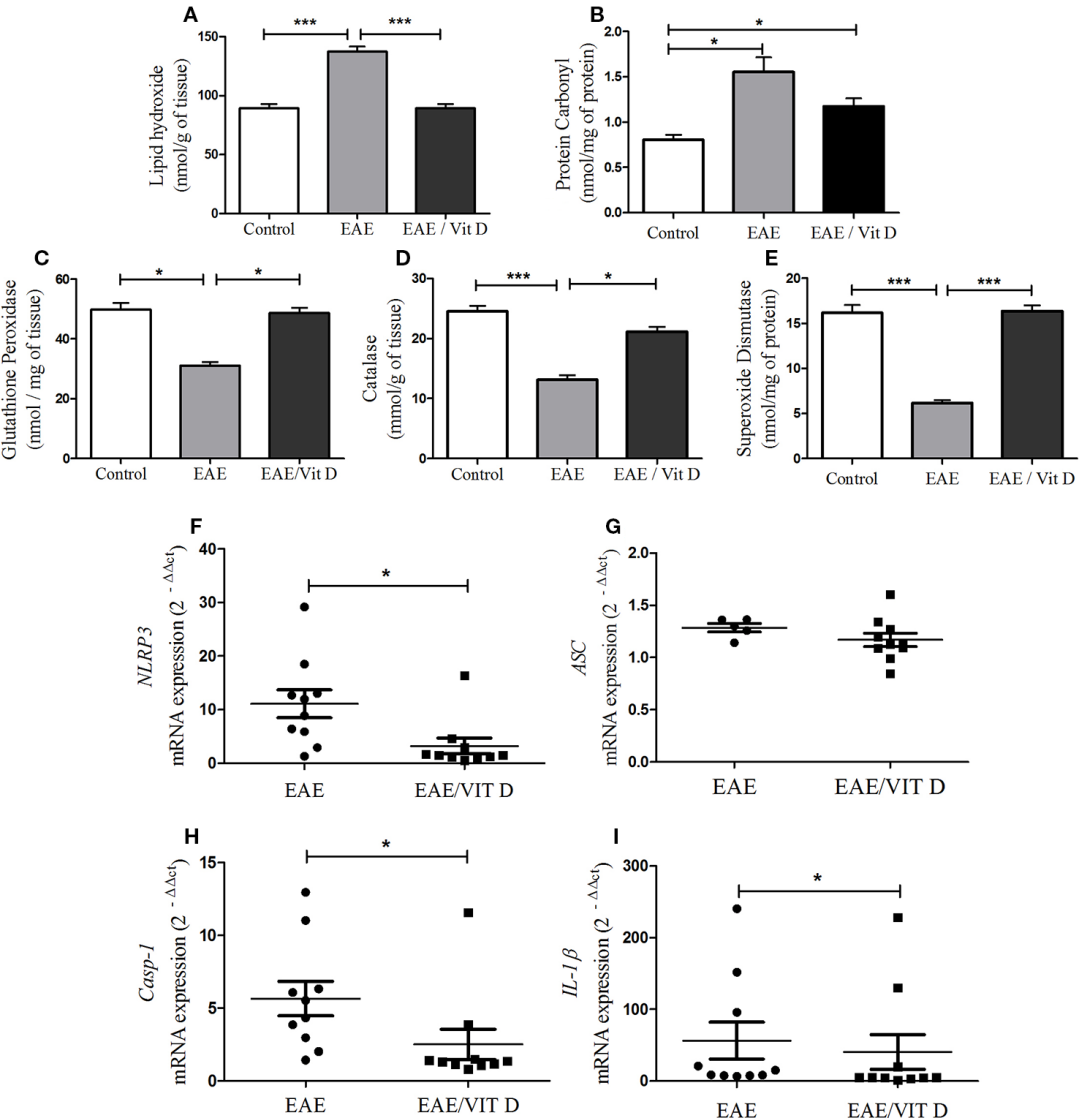
1,25-dihydroxyvitamin D3 reduces oxidative stress and inflammasome activation at the CNS. The CNS (brain and spinal cord) was collected from control, EAE, and EAE/Vit D mice previously perfused at the acute disease phase (18^th^ day). The brain and the cervical and thoracic portions of the spinal cord were pooled and used to evaluate oxidative stress status. Lipid hydroxide **(A)**, protein carbonyl **(B)**, the antioxidant enzymes glutathione peroxidase **(C)**, catalase **(D)**, and superoxide dismutase **(E)** were assessed by biochemical reactions. The lumbar spinal cord was used to determine the activation of the inflammasome. The expression level of mRNA for *NLRP3* **(F)**, *ASC* **(G)**, *caspase-1* **(H)**, and *interleukin (IL)-1β* **(I)** were determined by real-time PCR. Oxidative stress results were expressed as media ± SEM (8–11 animals/group). Results from two independent experiments were combined. ANOVA, Tukey's test. *p < 0.05 and ***p < 0.001. The qPCR results were expressed as median, 25–75% (box), and minimum-maximum (error bars) (10 animals/group). Results from two independent experiments were combined. Kruskal-Wallis, Dunn's test *p < 0.05.

As displayed in **Figures 6A, B**, the development of this pathology was concurrent with a conspicuous elevated level of lipid hydroxide (mean ± SEM): control: 89.3 ± 3.6, EAE: 137.1 ± 4.4, and EAE/Vit D: 89.3 ± 3.6; *f*: 51.0 and protein carbonyl (mean ± SEM): control: 0.8 ± 0.05, EAE: 1.5 ± 0.2, and EAE/Vit D: 1.2 ± 0.1; *f*: 9.8, thus revealing the existence of an accentuated oxidative stress condition. Concomitant and clear reductions were observed in the level of enzymes involved in the control of this oxidative stress, such as glutathione peroxidase (mean ± SEM): control: 49.8 ± 2.3, EAE: 31.0 ± 1.2, and EAE/Vit D: 48.6 ± 1.9; f: 35.8, catalase (mean ± SEM): control: 24.5 ± 0.9, EAE: 13.1 ± 0.8, and EAE/Vit D: 21.2 ± 0.8; f: 49.2 and superoxide dismutase (mean ± SEM): control: 16.2 ± 0.8, EAE: 6.1 ± 0.3, and EAE/Vit D: 16.3 ± 0.6; f: 92.0, as shown in **Figures 6C–E**, respectively. In these same illustrations, it can be observed that 1,25-VitD3 supplementation was able to counteract these pathological alterations.

As shown in **Figures 6F, H, I**, the administration of 1,25-VitD3 significantly reduced the gene expression of the inflammasome components *NLRP3*, *caspase-1*, and *IL-1β*, respectively. No change was detected in *ASC* expression (**Figure 6G**).

### Early Intervention With 1,25-VitD3 Restored Blood-Spinal Cord Barrier Permeability

**Figure 7:**
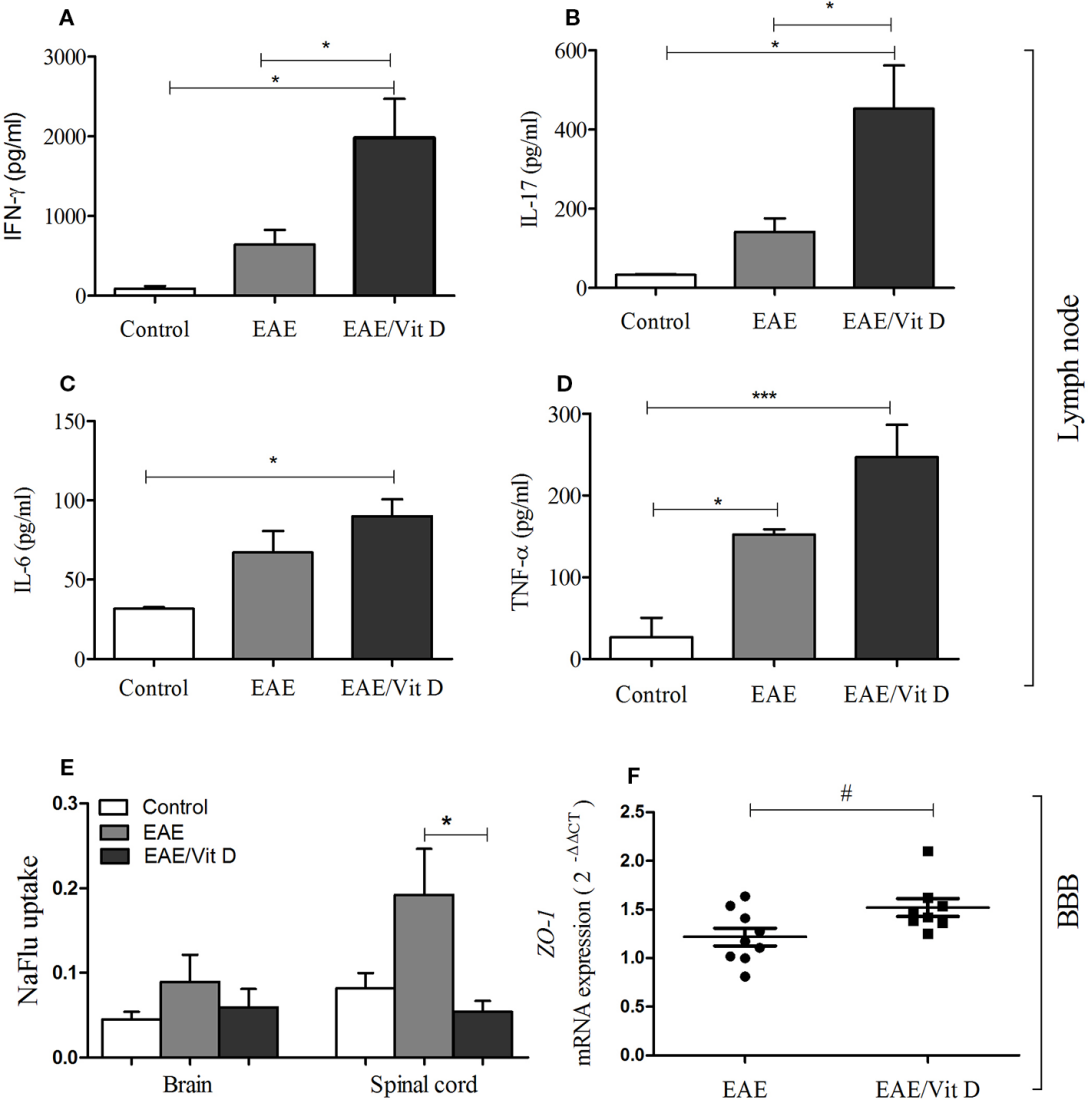
1,25-dihydroxyvitamin D3 preserved blood-spinal cord barrier permeability and triggered the accumulation of MOG responsive cells in the lymph nodes. Mice from control, EAE and EAE/Vit D were analyzed at two distinct time points to evaluate peripheral cytokine production, *ZO-1* mRNA expression level, and blood-CNS barrier permeability. Cytokine production by inguinal lymph node cells was tested at the acute disease phase (18^th^ day). Cell cultures were stimulated with MOG and levels of IFN-γ **(A)**, interleukin (IL)-17 **(B)**, IL-6 **(C)**, and TNF-α **(D)** were quantified in the supernatants by ELISA. The level of *ZO-1* mRNA expression at the lumbar spinal cord **(F)** was also tested on the 18^th^ day by PCR. The permeability tests **(E)** were performed at the preclinical disease phase (10^th^ day). Results were expressed as media ± SEM (9–15 animals/group). Results from two or three independent experiments were combined. ANOVA, Tukey's test or *t-test*, Mann-Whitney's test *p < 0.05, and ***p < 0.001. The qPCR result was expressed as median, 25–75% (box), and minimum-maximum (error bars) (nine animals/group). Results from two independent experiments were combined. Kruskal-Wallis, Dunn's test *p < 0.05 and ^#^p = 0.05.

To investigate whether the reduced neuroinflammation observed in mice given 1,25-VitD3 was associated with preservation of barrier permeability integrity between blood and CNS, we evaluated its permeability using the NaFlu test. As expected, barrier permeability was elevated in the brain (mean ± SEM): control: 0.05 ± 0.01, EAE: 0.09 ± 0.03, and EAE/Vit D: 0.06 ± 0.02; *f*: 3.1 and spinal cord (mean ± SEM): control: 0.08 ± 0.02, EAE: 0.19 ± 0.05, and EAE/Vit D: 0.06 ± 0.01; *f*: 3.1 of EAE mice, with spinal cord permeability being particularly disrupted (**Figure 7E**). 1,25-VitD3 preserved normal levels of permeability at the CNS, with the spinal cord normalized (**Figure 7E**). In line with these results, we also detected higher expression of *ZO-1* mRNA in lumbar spinal cord homogenates derived from 1,25-VitD3-injected mice (**Figure 7F**).

Cytokine production by cells from inguinal lymph nodes clearly indicated an accumulation or increased activation of MOG-responsive cells in 1,25-VitD3-supplemented animals. As presented in **Figures 7A–D**, EAE/Vit D animals produced significantly higher levels of IFN-γ (mean ± SEM): control: 88.6 ± 31.5, EAE: 640.7 ± 185.8, and EAE/Vit D: 1980 ± 487.5; *f*: 5.6, IL-17 (mean ± SEM): control: 33.2 ± 1.4, EAE: 142.1 ± 34.3, and EAE/Vit D: 452.7 ± 109.4; *f*: 6.8, IL-6 (mean ± SEM): control: 31.7 ± 1.0, EAE: 67.0 ± 13.7, and EAE/Vit D: 90.0 ± 10.6; *f*: 5.5, and TNF-α (mean ± SEM): control: 26.4 ± 24.1, EAE: 152.3 ± 6.6, and EAE/Vit D: 247.1 ± 39.3; *f*: 14.1, respectively.

## Discussion

The possibility of using active vitamin D3 as an alternative, adjunct, or even prophylactic therapy for MS has been investigated; however, it remains controversial. In this study, we used EAE, which is a widely adopted animal model, to evaluate the ability of this vitamin to control MS development, especially regarding the CNS. Our initial findings indicated that 1,25-VitD3 administration soon after disease induction was able to significantly arrest disease development by controlling the prevalence, disease severity, and the time required for the disease to manifest itself. All of these parameters are considered valid indicators of vitamin D or other drug efficacies in EAE (Mosayebi et al., 2006). The absence of body weight loss during therapies in this model is usually viewed as another indication of therapeutic efficiency (Gao et al., 2014). However, this parameter cannot be used when supraphysiological vitamin D doses are being administered since it is already known that this hormone triggers body weight loss in mice and humans (Missio et al., 2018; Khosravi et al., 2018). We believe that body weight loss is caused by both the anti-obesogenic property of vitamin D and its effect at the hypothalamus level. The anti-obesogenic effect of vitamin D is supported by experimental data from rodents (Sergeev, 2015; Nameni et al., 2017) and humans (Ljunghall et al., 1995). For example, it is known that calcitriol is able to strongly inhibit adipogenesis. The binding of calcitriol to VDR interrupts adipogenesis mainly by downmodulating the expression of C/EBP-β mRNA and protein (Blumberg et al., 2006). Moreover, recent data indicate that vitamin D can act directly in the hypothalamus, causing accentuated body weight reduction by lowering food consumption (Sisley et al., 2016). To date, there is no consensus concerning the amount of 1,25-VitD3 that should be prescribed to be therapeutic without being deleterious, and quite variable doses have been tested in both preclinical and clinical assays (Spach and Hayes, 2005; Häusler and Weber, 2019). The dose and therapeutic scheme employed in our investigation have also been adopted by other researchers (Lemire and Archer, 1991; Shirazi et al., 2017; Parastouei et al., 2018). Possible side effects such as hypercalcemia and body weight loss, which are commonly associated with supraphysiological doses, must certainly be carefully addressed in future investigations.

Histopathological analysis confirmed the protective effect of 1,25-VitD3 by revealing very discrete alterations at the CNS of EAE treated animals. This is in sharp contrast with the conspicuous inflammatory and demyelinating lesions that are considered hallmarks of this pathology (Gibson-Corley et al., 2016) and found in untreated EAE mice. The reduced local inflammation observed in supplemented mice was further confirmed by the quantification of cells eluted from the CNS. Flow cytometry analysis indicated that both lymphocytes and macrophages/activated microglia cell populations were significantly decreased in the EAE treated group. A similar reducing effect was not observed in normal mice supplemented with 1,25-VitD3 (data not shown). Quantification of T cell signature transcription factors indicated that *Th1* and *Th17* lymphocytes, identified by *Tbx21* and *RORc* mRNA expression, respectively, were significantly downmodulated by 1,25-VitD3_._ These findings are highly consistent with the pivotal role of these two T cell subsets in the immunopathogenesis of EAE and MS (Constantinescu et al., 2011). It is well established that Th1 cells act mainly through interaction with microglia and astrocytes by amplifying the inflammatory response, inhibiting the production of essential neurotrophic factors, and enhancing the recruitment of microglia and Th17 cells by the upregulation of essential chemokines (Prajeeth et al., 2017). These myelin-specific Th17 cells traffic to the CNS, where they secrete IL-17A which, through chemokine induction, attracts various immune cells (particularly myeloid cells) into the CNS, thereby initiating and perpetuating the inflammatory cascade (Rostami and Ciric, 2013).

Although a large body of experimental data has indicated that the presence of this vitamin provides an environment that facilitates *Foxp3* expression by T cells and the ensuing development of regulatory activity (Dankers et al., 2017), we found no increase in *Foxp3* mRNA expression at the spinal cord in mice that received this vitamin. However, *CX_3_CR1* and *CCL17* were downmodulated by 1,25-VitD3. Based on the investigation of Lampron et al. (2015), the reduction of *CX_3_CR1* mRNA was interpreted as an indication that phagocytosis of myelin debris, which is dependent on *CX_3_CR1* expression on microglia and its interaction with fractalkine, was no longer needed since myelin was no longer being destroyed. It is important to highlight that additional investigations support a neurotoxic contribution of CX3CL1 and CX3CR1 (Lauro et al., 2015). The lowered levels of CCL17 in the EAE/Vit D experimental group are also indicative of the described pathogenic role of this chemokine. CCL17 binds to its cognate chemokine receptor (CCR4) and drives the migration of DCs and CD4+T cells into the CNS during the effector phase of the disease (Ruland et al., 2017).

Other parameters, such as oxidative stress and inflammasome activation, were also downmodulated by 1,25-VitD3 administration. Notably, it has been well established that inflammation and oxidative stress, which mutually amplify each other, play a major role in the pathogenesis of MS and EAE (Ohl et al., 2016). 1,25-VitD3 also brought the oxidative stress markers (found altered in EAE mice) back to physiological levels; it normalized the elevated end products associated with oxidative stress (lipid hydroxide and protein carbonyl) and also the lowered antioxidant enzymes (superoxide dismutase, catalase, and glutathione peroxidase). This reduction in oxidative stress is likely due to the decreased presence of T cells in the CNS and the consequent lower interaction with innate immune cells. Two of the most studied innate cell populations involved in ROS-mediated tissue damage in MS are infiltrating macrophages and resident microglia (Chu et al., 2018). Interestingly, these two cell types expressed much lower levels of MHCII in 1,25-VitD3-treated mice, suggesting that they were less activated, which has previously been described by other authors (Kong et al., 2016; Zorzella-Pezavento et al., 2017). This decreased MHCII level is likely relevant for protection since the degree of recognition, proliferation, and activation of self-reactive T cells is highly dependent on the presentation of self-peptides by these APCs (Almolda et al., 2011).

The evidence obtained to date attributes an essential role in the activation of the inflammasome in EAE and MS development. This multiprotein complex is a powerful signaling platform that promotes T cell pathogenicity, CNS cell infiltration, and neurodegeneration (Martin, 2016). Our results indicated that 1,25-VitD3 was able to significantly control the local mRNA expression of *NLRP3*, *caspase-1*, and *IL-1β*, thus suggesting that the early intervention with 1,25-VitD3 reduced the activation of this pivotal amplifying proinflammatory platform. The essential contribution of this system to MS and EAE immunopathogenesis has been experimentally proven. For example, it has been shown that activated inflammasome components are present in MS lesions (Ming et al., 2002) and that genetic knockout for inflammasome components develop less severe EAE (Gris et al., 2010).

Based on literature data indicating that 1,25-VitD3 is able to suppress CD4+T cell proliferation and concomitantly increase PD-L1 expression, we expected that its protective effect in EAE was associated with the upregulation of PD-L1 expression. However, this possibility was not confirmed. This subject has not been deeply investigated, however our findings are congruent with the work of Dimitrov et al. (2017), who observed the increased expression of PD-L1 by 1,25-VitD3 in humans but not in mice.

Since the efficacy of some products to control EAE severity have been attributed or related to the maintenance of BBB integrity (Zhang et al., 2013; Wang et al., 2016; Hamana et al., 2017), we compared the degree of blood-CNS permeability among the three experimental groups. The NaFlu test clearly indicated that 1,25-VitD3 was preserving the integrity of this barrier at the spinal cord level. As alteration of the permeability of this barrier is critical for the entry of inflammatory cells into the CNS (Fabis et al., 2007; Bennett et al., 2010), this stabilization could contribute to the protective effect of vitamin D. BBB stabilization by 1,25-VitD3 was initially described in a mouse brain endothelial cell culture model by Won et al. (2015). More recently, using human endothelial cells, Takahashi et al. (2017), observed that these cells expressed VDR and that their contact with 1,25-VitD3 decreased permeability by reducing vascular cell adhesion molecule-1 expression and restoring the zonula occludens-1 and claudin-5. Since the tight junction protein ZO-1 controls endothelial adherens junction, cell-cell tension, angiogenesis, and barrier formation (Tornavaca et al., 2015), we also evaluated *ZO-1* mRNA expression in the lumbar spinal cord. Interestingly, the level of mRNA *ZO-1* expression was significantly elevated in the supplemented EAE group. To the best of our knowledge, BBB stabilization by 1,25-VitD3 at the level of the spinal cord in EAE mice is being described here for the first time. However, it remains important to remember that many other molecules are part of this barrier and that their expression could also be modulated by 1,25-VitD3, such as claudins and occludin (Stamatovic et al., 2016; Takahashi et al., 2017). Interestingly, the stabilization of this barrier coincided with a clear accumulation of MOG-reactive cells at the draining lymph nodes, which was detected by the increased production of proinflammatory cytokines by these cells in the presence of MOG. This finding is in contrast with the downmodulatory effect of vitamin D in cytokine production by spleen cells from normal mice (Mimura et al., 2016; Missio et al., 2018). A possible explanation for these findings is that vitamin D effects are distinct in normal and EAE conditions. Notably, EAE induction is a very stringent process that requires CFA and pertussis toxins. This vigorous proinflammatory condition could counteract the downmodulatory effect of this vitamin. In addition, the stabilization of BBB by 1,25-VitD3 is likely contributing to the accumulation of MOG-reactive cells at the lymph nodes.

We believe that the aforementioned alterations are either directly or indirectly involved in the protective 1,25-VitD3 effect characterized in this work. This 1,25-VitD3 property is probably secondary to the suppression of encephalitogenic Th response. This possibility is highly strengthened by the seminal findings of Hayes’ research team (Hayes et al., 2015). By employing bone marrow chimeric mice displaying a disrupted VitD3 receptor (VDR) in hematopoietic and non-hematopoietic cells, they initially revealed that EAE inhibition by this hormone was dependent upon VDR activity in hematopoietic cells. They further disclosed that this inhibition occurred by a direct effect on Th1 and Th17 cells (Mayne et al., 2011). Interestingly, they had previously shown that 1,25-VitD3 activity over Th1 cells occurs at the CNS and not at the periphery, as might be expected (Nashold et al., 2001). As such, our results can be better understood in the context of these cited works. For example, we also observed no inhibition of Th1 and Th17 cell priming and cytokine production at the periphery. Moreover, a very reduced number of infiltrating cells and lower macrophage and microglia activation were found in the CNS of these supplemented mice. These findings could be secondary to the death of effector T cells by CNS-derived apoptotic signals, as has been previously reported (Nataf et al., 1996; Hayes et al., 2015).

Together with the literature data, this work reinforces the ability of the active form of vitamin D to control EAE development. Overall, our results indicate that a precocious and constant supply of supraphysiological levels of this hormone is able to prevent pivotal events in the CNS that are involved in the immunopathogenesis of this disease, such as disruption of the blood-spinal cord barrier permeability, inflammatory cell accumulation and activation, oxidative stress, and inflammasome activation.

Taken together, these findings contribute to establishing the necessary knowledge to consider this hormone as a possible prophylactic and/or therapeutic alternative in MS management.

## Data Availability Statement

The raw data supporting the conclusions of this article will be made available by the authors, without undue reservation, to any qualified researcher.

## Ethics Statement

This study was approved by the Ethics Committee on Animal Experimentation of the Institute of Biosciences, Unesp, Botucatu (CEUA-Process 926).

## Author Contributions

LO, SZ-P, and AS contributed to the conception and design of this work. LO, SZ-P, TF-S, LM, LI, and AF performed experiments. LO, TF-S, LM, and LI contributed to data acquisition. LR, TF-S, LM, LI, and AS contributed to data interpretation. LR and AS wrote the paper and all authors reviewed the manuscript and approved the final version.

## Funding

This work was supported by São Paulo Research Foundation-FAPESP (grant number 2013/26257-8) and by the National Council for Scientific and Technological Development – CNPq (grant number 307269/2017-5).

## Conflict of Interest

The authors declare that the research was conducted in the absence of any commercial or financial relationships that could be construed as a potential conflict of interest.
